# Intraspecific variation in wing geometry among *Tabanus rubidus* (Diptera: Tabanidae) populations in Thailand

**DOI:** 10.3389/fvets.2022.920755

**Published:** 2022-09-02

**Authors:** Tanawat Chaiphongpachara, Thekhawet Weluwanarak, Tanasak Changbunjong

**Affiliations:** ^1^Department of Public Health and Health Promotion, College of Allied Health Sciences, Suan Sunandha Rajabhat University, Bangkok, Thailand; ^2^The Monitoring and Surveillance Center for Zoonotic Diseases in Wildlife and Exotic Animals, Faculty of Veterinary Science, Mahidol University, Nakhon Pathom, Thailand; ^3^Department of Pre-clinic and Applied Animal Science, Faculty of Veterinary Science, Mahidol University, Nakhon Pathom, Thailand

**Keywords:** geometric morphometrics, horse flies, landmark, Tabanidae, *Tabanus rubidus*

## Abstract

*Tabanus rubidus* (Wiedemann, 1821) (Diptera: Tabanidae) is a hematophagous insect of veterinary and medical importance and is the predominant *Tabanus* spp. in Thailand. It is a potential mechanical vector of *Trypanosoma evansi*, which causes surra in domestic and wild animals. Wing geometric morphometrics is widely used as morphological markers for species identification and to assess the insect population structure. Herein, we investigated the intraspecific variation in wing geometry among *T. rubidus* populations in Thailand using landmark-based geometric morphometric analysis. *Tabanus rubidus* females were collected from five populations in four geographical regions in Thailand. The left wings of 240 specimens were removed and digitized using 22 landmarks for analysis. While wing size variations were found between some populations, wing shape variations were detected in all. These intraspecific variations in *T. rubidus* populations indicate an adaptive response to the local environmental conditions.

## Introduction

Hematophagous dipterans or blood-sucking insects are major causes of veterinary and medical diseases worldwide, as they carry and transmit several pathogens to animals and humans (1). Horse flies (*Tabanus* spp.), an important blood-sucking insect species, are members of the family Tabanidae, with approximately 4,400 known species (2), of which 1,300 are in the genus *Tabanus* (3). As adult female horse flies require blood meals for egg production and development, they cause severe irritation, stress, blood loss, reduced feed intake, decreased weight, and decreased milk production in livestock, leading to economic losses (2, 4). They are major mechanical vectors of pathogens including viruses, bacteria, protozoa, and helminths in livestock, pets, and wildlife (2, 4), and can also sometimes transmit these to humans (5). Adult male horse flies cannot transmit diseases (non-vector) as they are not hematophagous and only feed on flower nectar or natural sugar sources (2). It is crucial to understand the biology of horse flies in the natural environment in order to control their population in the target areas.

Thailand is a tropical country where diverse species of horse flies are abundantly found (45 reported species) (6) with *Tabanus rubidus* (Wiedemann, 1821) being most predominant (6, 7). This species was confirmed to be mechanical vectors of pathogens like *Clostridium chauvoei* (causing gas gangrene) and *Bacillus anthracis* (causing anthrax) (2). Additionally, it is also suspected to transmit *Trypanosoma evansi*, which causes trypanosomosis (surra) in horses, cattle, and buffaloes (5, 8). Recently, Changbunjong et al. (6) surveyed *Tabanus* species in different habitats in Thailand and reported that *Tabanus striatus* (25.45%), followed by *Tabanus megalops* (21.36%), *T. rubidus* (14.82%), *Tabanus tamthaiorum* (7.90%), and *Tabanus oxybeles* (6.38%), are the five most abundant species. Moreover, they also revealed that the abundance of horse flies depends on the geographical areas, consistent with other studies showing relationship between environmental and geographical conditions and the number of horse flies (9), and that some morphological features may be linked to environmental adaptation (10).

It is imperative to understand how different areas affect insect vectors to control and monitor their natural populations as some have disease transmission-related behaviors between areas. For example, Wamaket et al. (11) surveyed the behavior of *Anopheles* mosquitoes as malaria vectors in many areas of southern Thailand, and found that each species has different bite times in different areas. Furthermore, many vector species also display phenotypic trait variations within species (intraspecific variation) (12).

Intraspecific variation arising from genetic and phenotypic diversity within and among populations is essential for adaptation in response to different environmental conditions (13). For instance, morphological characteristics, such as the forewing and hindwing sizes of grasshoppers (*Trilophidia annulata*), varied among populations depending on ecological, climatic, and geographical factors (14). Wing geometry, including size and shape, is a good indicator to investigate phenotypic adaptations to specific environments (15). The insect size varies highly based on environmental conditions (16). A larger wing size in insects is associated with longer life spans, allowing them to spread pathogens for a long period of time in nature (17). As for wing shape of insects, it is a recognized, species-specific characteristic associated with genetic background (18). Moreover, Morales Vargas et al. (19) demonstrated that variations in wing shape indicate geographic differences.

Wing geometric morphometrics is an effective tool used to examine variability in wing size and shape of insects (20, 21). This technique was used to study the intraspecific variation for insect vectors such as mosquitoes [*Anopheles* (*Cellia*) *epiroticus*] (22, 23), sand flies (*Phlebotomus stantoni* and *Sergentomyia hodgsoni*) (24), stable flies (*Stomoxys calcitrans*) (25), tsetse flies (*Glossina palpalis*) (26), and horse flies (*Tabanus bromius*) (27). However, the microgeographic wing variation of *T. rubidus* remains unknown, although it is the dominant species in several countries including Thailand.

Herein, we used landmark-based geometric morphometrics to examine intraspecific variation in wing size and shape among five populations of *T. rubidus* in Thailand. Our results on wing variations can elucidate population morphological dynamics and microevolution patterns of this vector to develop effective control measures.

## Materials and methods

### Ethical considerations

This study was reviewed and approved by the Animal Care and Use Committee, Faculty of Veterinary Science, Mahidol University, Thailand (Ethics Approval Number: MUVS-2020-01-01).

### Specimen collection and species identification

**Figure 1:**
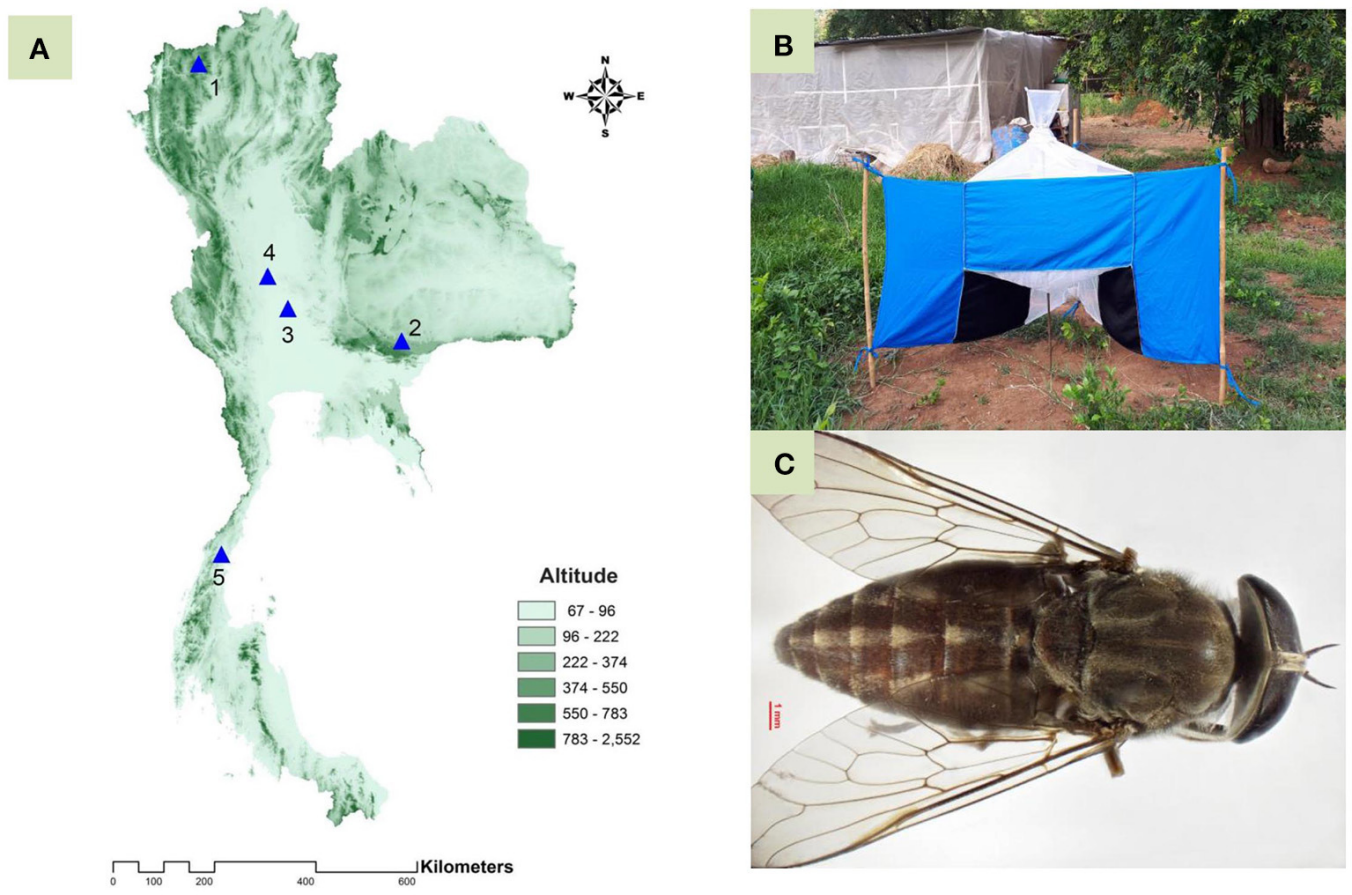
Topographic map of Thailand showing five populations of *Tabanus rubidus*: 1, Chiang Mai; 2, Nakhon Ratchasima; 3, Uthai Thani; 4, Singburi; and 5, Chumphon **(A)**; Nzi Trap used for collecting specimens from genus *Tabanus* **(B)**; and adult *T. rubidus* female specimen **(C)**.

A total of five populations of *T. rubidus*, representing four different geographical regions in Thailand—Chiang Mai (Northern region), Nakhon Ratchasima (Northeastern region), Uthai Thani (Central region), Singburi (Central region), and Chumphon (Southern region)—were selected based on a report by Changbunjong et al. (28) (Figure 1A and Table 1). Adult female horse flies were collected using five Nzi Traps (29) (Figure 1B) per population for two consecutive days from 06:00 to 18:00 h between February and November 2020. Flies were collected at 2 or 3 h intervals to prevent specimen damage. They were euthanized by freezing at −10°C, placed individually in 1.5 ml microcentrifuge tubes, labeled by their population, and brought back to the Vector-Borne Diseases Research Unit, Faculty of Veterinary Science, Mahidol University, Nakhon Pathom, Thailand. Furthermore, *T. rubidus* specimens (Figure 1C) were identified based on morphological characters using the taxonomic key by Burton (30) under a stereomicroscope (Nikon AZ 100, Nikon Corp, Tokyo, Japan) and stored at −20°C for further specimen preparation.

**Table 1:** Details of population, month, and number (*n*) of female *Tabanus rubidus* specimens used for the landmark-based geometric morphometric analysis.

**Population**	**Code**	**Region**	**Month**	**Characteristic of collection area**	**Altitude (m)**	**Latitude/Longitude**	***N***
Chiang Mai	CM	Northern	Apr	Beef cattle farm located in rural area	624	N19°22′42″, E098°43′25″	40
Nakhon Ratchasima	NR	Northeastern	Feb	Buffalo farm located in rural area	498	N14°16′54″, E102°28′16	50
Uthai Thani	UT	Central	Nov	Beef cattle farm located in rural area	38	N15°24′13″, E100°00′49″	50
Singburi	SB	Central	Aug	Beef cattle farm located in rural area	18	N14°54′59″, E100°22′38″	50
Chumphon	CP	Southern	Feb	Beef cattle farm located in rural area	14	N10°29′33″, E099°08′28″	50

### Specimen preparation and data collection

**Figure 2:**
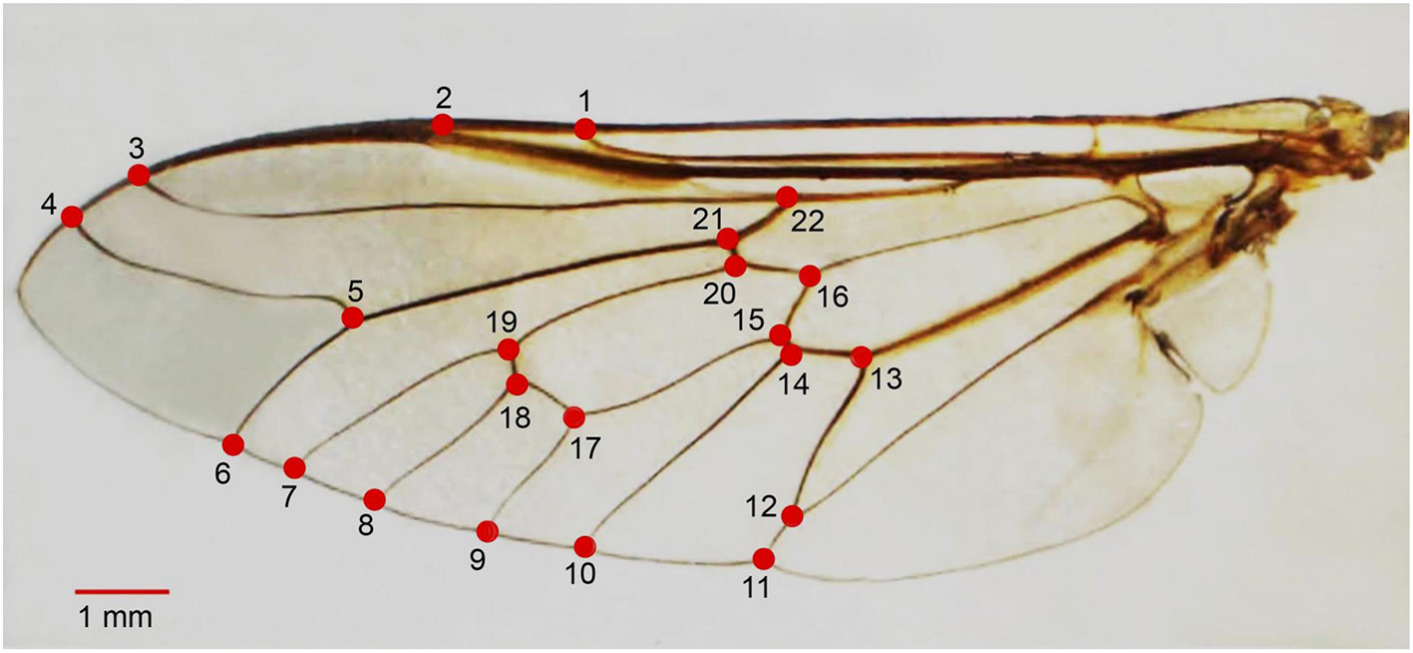
Position of the 22 landmarks on the left wing of adult female *Tabanus rubidus* used for geometric morphometric analysis.

Each left wing from an adult female *T. rubidus* was detached from the thorax using a sterilized blade and mounted on a microscope slide using Hoyer's mounting medium. Further, each wing slide was photographed with a digital camera connected to a stereomicroscope (Nikon AZ 100, Nikon Corp, Tokyo, Japan) and a 1-mm scale unit was added to each wing image. All left wing images were digitized based on coordinates of 22 landmarks covering all intersections of wing veins (31) (Figure 2). Landmark digitization, geometric morphometric analyses, and graphical outputs were performed using XYOM online tool (32) (https://xyom.io/me).

### Repeatability and allometry

To precisely digitize the coordinate landmarks, 20 *T. rubidus* wings were randomly selected and digitized twice by the same person (repeatability test). The measurement error of landmark digitization was estimated by the repeatability index, which computed based on the Procrustes analysis of variance (ANOVA) method, as described by Arnqvist and Mårtensson (33). To assess the effect of wing size related wing shape variation (allometry), we used linear regression based on discriminant factor (DF) (shape variable) and centroid size (CS) (size variable) and evaluated using the determination coefficient (*r*^2^).

### Wing size analyses

Overall wing size (also called global size) of *T. rubidus* was estimated by the CS as described by Bookstein (34). The wing CS variation among *T. rubidus* populations were illustrated using box plots. The average wing CS differences between populations were performed using one-way ANOVA followed by Bonferroni *post-hoc* test. The statistical significance was estimated using a non-parametric permutation test (1,000 permutations) at *p*-value < 0.05.

### Wing shape analyses

To extract wing shape variables, each landmark dataset was superimposed using Generalized Procrustes Analysis. The principal components of wing shape variables were used as final variables. Visual comparison of shape changes across populations was obtained by superposing average wing of each population. The final wing shape variables were used as input for discriminant analysis (DA), represented by the factor map. Moreover, Mahalanobis distances were calculated to estimate the metric distance between *T. rubidus* populations. The non-parametric permutation test (1,000 permutations) was used to calculate the statistical significance in Mahalanobis distance differences among populations (*p*-value < 0.05).

### Relationships of wing shape among populations

The UPGMA (Unweighted Pair Group Method with Arithmetic Mean) tree based on the Euclidean distances was used to illustrate the pattern of relationship between wing shapes among populations. To determine branch reliability, each branch support was estimated, based on 1,000 bootstrap replicates.

### Validated classification

Cross-validated classification (leave-one-out cross-validation) was used to determine the percentage of specimens correctly classified within their populations. Each specimen was sequentially removed from the total specimens and assigned to the most likely (size) or closest (shape) group, based on the maximum likelihood approach and Mahalanobis distance, respectively.

## Results

### Repeatability and allometry

**Figure 3:**
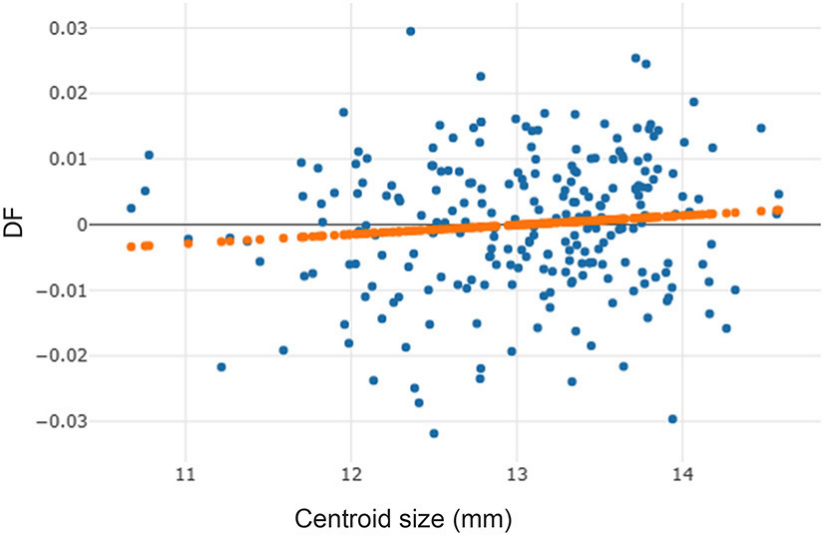
Linear regression prediction (orange dots line) between wing centroid size (horizontal axis) and discriminant factor (DF) (vertical axis) of *Tabanus rubidus* specimens.

The high repeatability score for wing shape (94%) indicated that our coordinate plotting in the image set was highly accurate. The measurement error for the comparison of landmark digitization in the wing image set was relatively low (6%). The allometric effect of *T. rubidus* specimens was very low (*r*^2^ = 1%, Figure 3) and not statistically significant (*p* > 0.05). Thus, the wing size changes of *T. rubidus* were not affected by wing shape changes.

### Wing size variation

**Figure 4:**
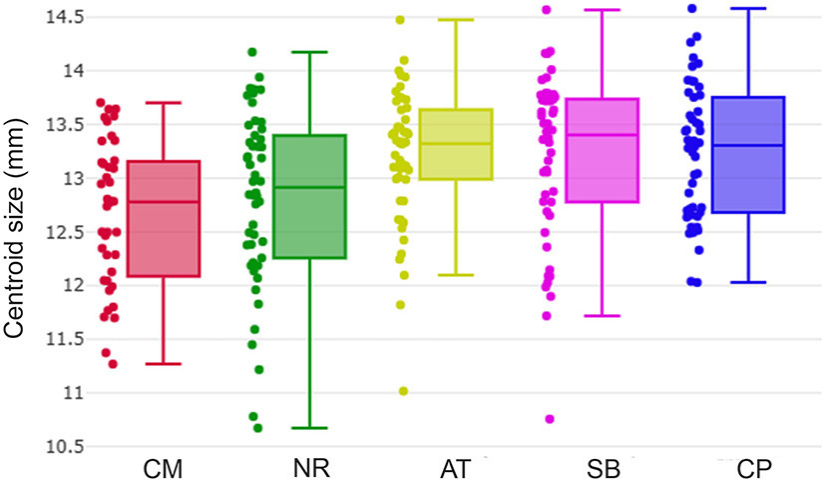
Wing centroid size variation of *Tabanus rubidus* among populations (CM, Chiang Mai; NR, Nakhon Ratchasima; AT, Uthai Thani; SB, Singburi; CP, Chumphon). Each box plot displays the median with the 25^th^ and 75^th^ quartiles. Dots along the sides of each box represent the individual sizes.

The *T. rubidus* wing CS variation among populations is shown in Figure 4. Mean wing CS of adult *T. rubidus* populations ranged from 12.67 to 13.23 mm (Table 2), with the highest mean wing CS seen in *T. rubidus* from Chumphon (13.23 mm), while the lowest mean wing CS was seen in *T. rubidus* in Chiang Mai (12.67 mm). The remaining *T. rubidus* populations had the following mean wing CS: 12.81 mm (Nakhon Ratchasima), 13.19 mm (Uthai Thani), and 13.20 mm (Singburi). The statistical significances in difference in mean wing CS of *T. rubidus* among populations are shown in Table 3.

**Table 2:** Mean wing centroid size of *Tabanus rubidus* populations.

**Population**	***N***	**Mean (mm)**	**(Min–Max)**	**Variance**	**SD**	**SE**
CM	40	12.67	11.27–13.70	0.46	0.68	0.11
NR	50	12.81	10.67–14.17	0.67	0.82	0.12
AT	50	13.19	11.02–14.47	0.40	0.63	0.09
SB	50	13.20	10.76–14.57	0.60	0.77	0.11
CP	50	13.23	12.03–14.58	0.39	0.62	0.09

N, number; Min, minimum; Max, maximum; SD, standard deviation; SE, standard error; CM, Chiang Mai; NR, Nakhon Ratchasima; AT, Uthai Thani; SB, Singburi; CP, Chumphon.

**Table 3:** Pairwise significant differences among the wing centroid size of *Tabanus rubidus* populations.

**Population**	**CM**	**NR**	**AT**	**SB**	**CP**
CM	–				
NR	N	–			
AT	S	S	–		
SB	S	S	N	–	
CP	S	S	N	N	–

N, not significant (*p* > 0.05); S, significant (*p* < 0.05); CM, Chiang Mai; NR, Nakhon Ratchasima; AT, Uthai Thani; SB, Singburi; CP, Chumphon.

### Wing shape variation

**Figure 5:**
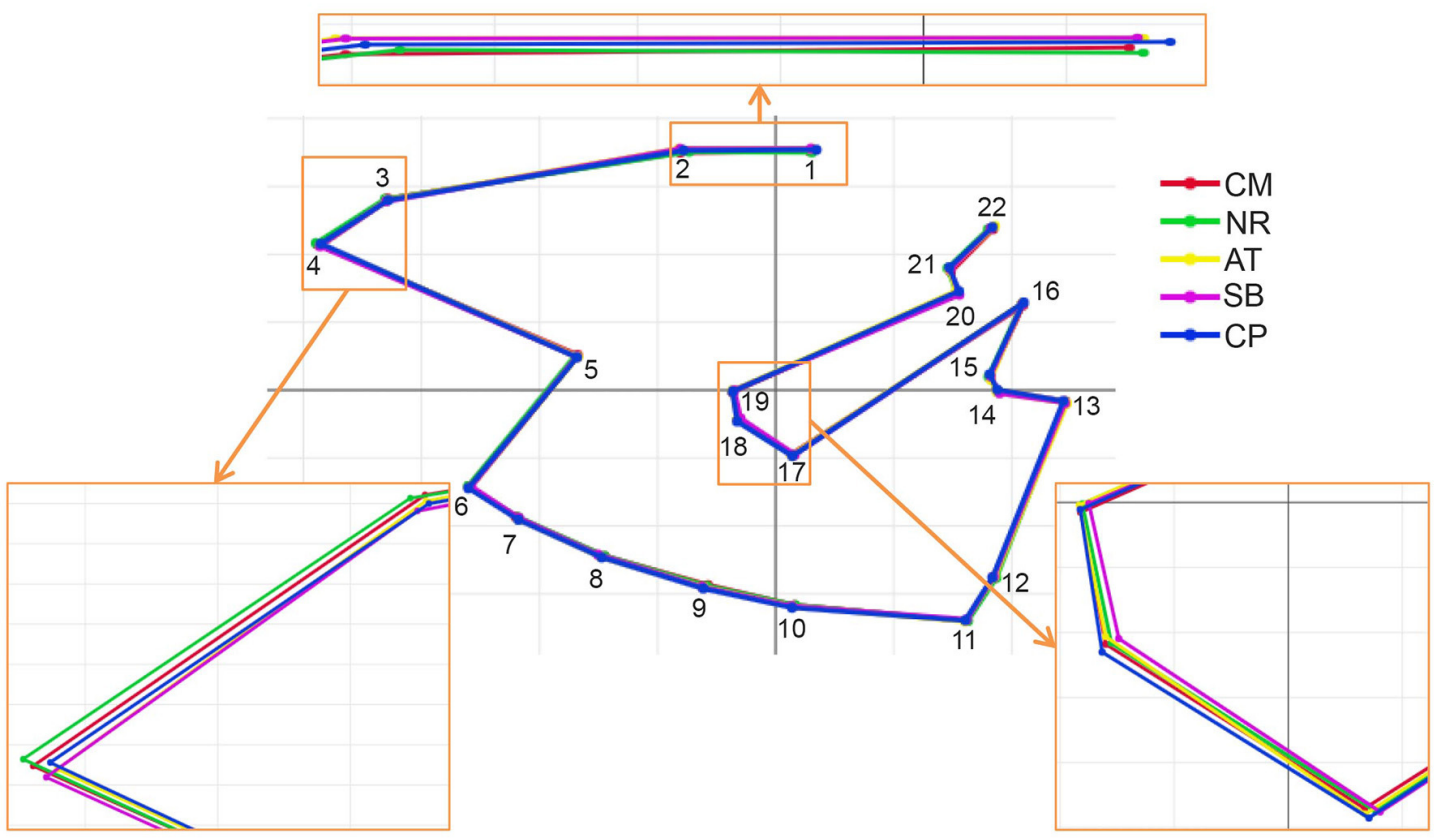
Superposition of the mean shape landmark configurations of *Tabanus rubidus* among populations (CM, Chiang Mai; NR, Nakhon Ratchasima; AT, Uthai Thani; SB, Singburi; CP, Chumphon). Small frames show enlarged images of wing shape with variations.

**Figure 6:**
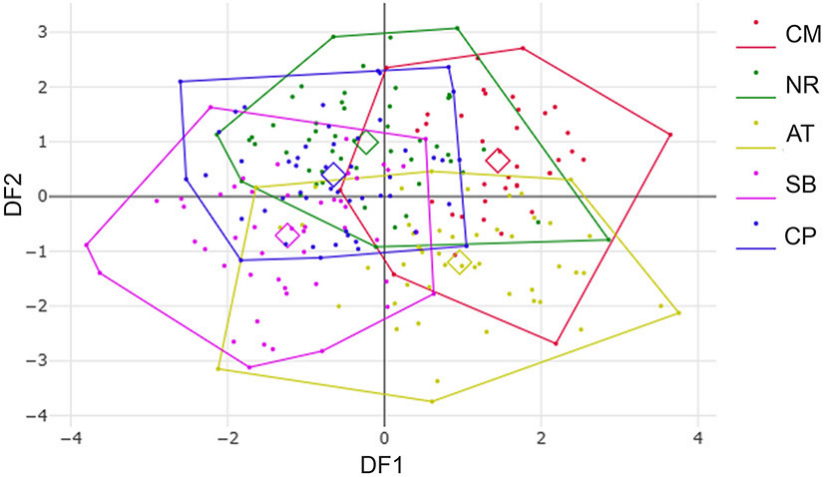
Factor map based on discriminant analysis showing wing shape divergence of *Tabanus rubidus* among populations (CM, Chiang Mai; NR, Nakhon Ratchasima; AT, Uthai Thani; SB, Singburi; CP, Chumphon). Each point in each population polygon represents an individual specimen with the small squares representing a mean group. The horizontal axis was the first discriminant factor (DF1), while the vertical axis was the second discriminant factor (DF2).

After the Generalized Procrustes Analysis, graphic superimposition of the mean landmark configuration was constructed to provide shape line differences in the average wing of each population. Graphic superimposition of landmark coordinates revealed significant displacement at 1, 2, 3, 4, 17, 18, and 19 landmark positions when comparing among populations (Figure 5). The factor map, based on DA, is shown in Figure 6, and indicated that all populations of *T. rubidus* were markedly overlapped. This map was defined by the first two DF axes, which accounted for 75.4% of the total shape variation for *T. rubidus* (DF1 = 43.2% and DF2 = 32.2%). The pairwise Mahalanobis distances were significantly different among the populations (*p* < 0.05, Table 4).

**Table 4:** Mahalanobis distances among the wing shape of *Tabanus rubidus* populations.

**Population**	**CM**	**NR**	**AT**	**SB**	**CP**
CM	–				
NR	2.00	–			
AT	2.13	2.60	–		
SB	3.04	2.12	2.45	–	
CP	2.56	1.91	2.49	2.14	–

All pairwise Mahalanobis distances were significant differences at *p* < 0.05. CM, Chiang Mai; NR, Nakhon Ratchasima; AT, Uthai Thani; SB, Singburi; CP, Chumphon.

### Wing shape relationships and validated classification

**Figure 7:**
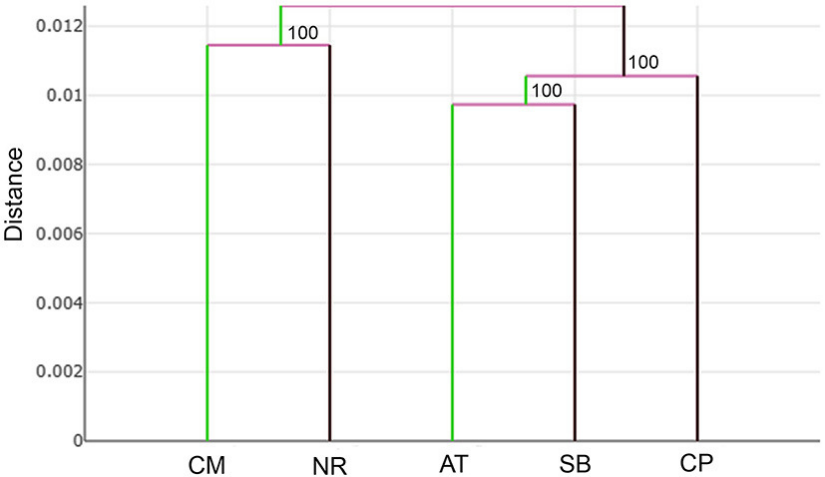
UPGMA tree of *Tabanus rubidus* wing shape among populations (CM, Chiang Mai; NR, Nakhon Ratchasima; AT, Uthai Thani; SB, Singburi; CP, Chumphon) based on the Euclidean distances with 1,000 bootstrap replicates.

The UPGMA tree based on the Euclidean distances of *T. rubidus* among populations showed the proximity of wing shape between five *T. rubidus* populations supported by high bootstrap values (Figure 7). The *T. rubidus* wing shape of the Uthai Thani population was more similar to Singburi population than Chumphon population, while the Chiang Mai population was similar to Nakhon Ratchasima population, and was separated from other populations. Validated scores of the classification of *T. rubidus* among populations revealed total performance of 24.58% for wing size (ranged from 2 to 58%) and 65.42% for wing shape (ranged from 58 to 70%) (Table 5).

**Table 5:** Scores of validated classification based on the maximum likelihood for wing size and Mahalanobis distance for wing shape among *Tabanus rubidus* populations.

**Population**	**Wing size**		**Wing shape**	
	**% correctly assigned individuals**	**No. of correctly assigned individuals/Total numbers**	**% correctly assigned individuals**	**No. of correctly assigned individuals/Total numbers**
CM	20	8/40	65	26/40
NR	28	14/50	58	29/50
AT	2	1/50	70	35/50
SB	58	29/50	66	33/50
CP	14	7/50	68	34/50
Total	24.58	59/240	65.42	157/240

CM, Chiang Mai; NR, Nakhon Ratchasima; AT, Uthai Thani; SB, Singburi; CP, Chumphon.

## Discussion

To our knowledge, this study is the first to report the intraspecific variation in wing geometry among *T. rubidus* populations based on geometric morphometrics. The results showed that wing size variations were observed between some populations, while wing shape variations were detected among populations, indicating that wing geometry differs according to geographical environment.

Linear regression analysis based on CS on shape variables, conducted to show the statistical relationship between size and shape (allometry), revealed that there was no significant relationship between the wing size and shape of *T. rubidus* specimens. Therefore, the differences in wing size were not affected by wing shape variation. Although correlations between wing size and shape have been found in many insects like blow flies (35), stable flies (36), mosquitoes (37), *Haematobosca aberrans* (38), and other *Tabanus* spp. (31), a few did not display this relationship due to variations caused by evolutionary divergence (20). Some studies have explored the influence of allometry on the geometric morphometric analysis as it is possible that an allometric residue remained in the shape variables (39). However, the results of our study showed that the specimens were not influenced by allometric effect. Hence, we did not remove this effect while analyzing wing shape variation.

The intraspecific variation in the wing size of *T. rubidus* populations in Thailand confirmed that Chumphon population had the largest size, while Chiang Mai population had the smallest. Environmental conditions, such as temperature, larval density, food quality, and food quantity, influence the wing size of insects during the immature stages (40). Recently, Baleba et al. (36) studied the changes in *S. calcitrans* wings and found that lowest larval density and good substrate quality resulted in the largest wing size in adult flies. Furthermore, large wing size can be linked to abundance of favorable environmental conditions. Accordingly, it is highly probable that the environmental conditions especially temperature and larval diet in Chumphon, Southern Thailand, are suitable for the development of immature *T. rubidus*, while those of Chiang Mai, Northern Thailand, might be comparatively less suitable. However, the wing size was more sensitive to environmental changes than wing shape (15). In this study, as we collected specimens for each population at different periods between February and November 2020, the wing size might possibly be influenced by the seasonality. Thereby, wing shape, which highly stable to climatic factors (19), is more appropriate for examining intraspecific variation among populations than wing size.

Although the factor maps based on DA did not show clear segregation among the five populations, wing shape variation was detected in *T. rubidus* in these populations by comparing pairwise Mahalanobis distances. Mahalanobis distance is an effective statistical technique to measure the metric distance between a point and a distribution, and is widely used for estimating differences of intra- and interpopulation variations in wing shape of insect vectors (15, 39, 41). Intraspecific variation in insect wing shape is caused by many different factors, such as genetic background (42), conditions of larval habitats (36), and altitudes (27).

The differences in *T. rubidus* wing shapes among populations can be attributed to their adaptation to local environmental conditions. Wing shape changes affect their flight performance, which is linked to the host-seeking behavior of vectors (15, 43). The UPGMA tree, which shows *T. rubidus* wing shape relationships among populations, revealed that Chiang Mai and Nakhon Ratchasima populations were similar and separated from the remaining populations. The Uthai Thani population was more similar to Singburi than to the Chumphon population. This pattern of the topology clearly indicates that *T. rubidus* wing shape is related to altitude. The *T. rubidus* populations in high altitude areas, including Chiang Mai (624 m) and Nakhon Ratchasima (498 m), are distinctly separated from the low altitude areas including Uthai Thani (38 m), Singburi (18 m), and Chumphon (14 m). This result is consistent with the *T. bromius* populations studied by Altunsoy et al. (27). They found that altitude difference affects wing shape of this *Tabanus* species. Similarly, a previous study by Kuclu et al. (44) showed that altitude influenced the wing shapes of *Aedes vexans* in northeastern Turkey. Altitudinal gradients can act as biological models to study the impact of increase in environmental factors, such as temperature, atmospheric pressure, levels of sunlight, vegetation cover, and relative humidity, on biodiversity (45). Several studies investigated the effect of altitude and landscape structure on horse flies and revealed that altitude influenced species richness and abundance (46–48). Differences of ecosystems in each altitude level might be the main factor causing changes in *T. rubidus* wing shape reflecting environmental adaptation patterns.

The results of validated classification revealed that the wing shapes were more specific to the geographical populations (65.42% total performance score) than wing size (24.58%). This indicated that spatial differences affect the altered wing shape of female *T. rubidus*. A previous study on phenetic structure of *Aedes albopictus* populations demonstrated that wing shape was an important variable to indicate heritable intraspecific and geographic differences (19). Although intraspecific variation depends largely on environmental factors, it can also result from genetic factors (20). However, a study on genetic differences based on cytochrome *c* oxidase subunit I in *T. rubidus* populations from six locations of Thailand—Chiang Mai, Nakhon Ratchasima, Uthai Thani, Chumphon, Kanchanaburi, and Chainat Provinces—showed low intraspecific divergence, ranging from 0 to 1.9% (mean = 0.9%) (28). Therefore, these results indicated that intraspecific variation of wing geometry of the *T. rubidus* population seen in this study might be less related to genetic variation of this species. A better understanding of population structure of *T. rubidus* is essential for developing effective population control. For example, an insecticide application could be sequential in case of separation between populations because these populations may respond differently to use of a same insecticide. However, the relationship between intraspecific morphological variation and insecticide resistance should be considered in further studies.

## Conclusions

In the present study, geometric morphometrics was used to investigate the intraspecific variation of wing geometry among *T. rubidus* populations in Thailand. Our results revealed wing size variations between some populations of *T. rubidus*, while wing shape variations were detected in all. Wing size variation did not have significant effect on wing shape variation. These results indicated that the wing shape of *T. rubidus* populations is an adaptive response to local environmental pressures in the studied geographical areas. Altitude was implicated as an important factor for this variation. Our results might enable better understanding of the population structure of *T. rubidus* in Thailand for developing effective population control.

## Data availability statement

The original contributions presented in the study are included in the article/supplementary material, further inquiries can be directed to the corresponding author/s.

## Ethics statement

The animal study was reviewed and approved by the Animal Care and Use Committee, Faculty of Veterinary Science, Mahidol University, Thailand (Ethical Approval Number: MUVS-2020-01-01).

## Author contributions

TChai and TChan: conceptualization, methodology, data curation, and writing-original draft preparation. TChai, TChan, and TW: validation, investigation, and writing-review and editing. TChan: resources, project administration, and funding acquisition. All authors have read and agreed to the published version of the manuscript.

## Funding

This study was financially supported by the Faculty of Veterinary Science, Mahidol University.

## Conflict of interest

The authors declare that the research was conducted in the absence of any commercial or financial relationships that could be construed as a potential conflict of interest.

## Publisher's note

All claims expressed in this article are solely those of the authors and do not necessarily represent those of their affiliated organizations, or those of the publisher, the editors and the reviewers. Any product that may be evaluated in this article, or claim that may be made by its manufacturer, is not guaranteed or endorsed by the publisher.
